# Guillain-Barré Syndrome Presenting as an Acute Back Pain

**DOI:** 10.7759/cureus.17540

**Published:** 2021-08-29

**Authors:** Nicholas T Hodgeman, Lacy E Lowry, Sky D Graybill

**Affiliations:** 1 Department of Medicine, San Antonio Uniformed Services Health Education Consortium, San Antonio, USA; 2 Department of Internal Medicine, San Antonio Uniformed Services Health Education Consortium, San Antonio, USA; 3 Department of Endocrinology, San Antonio Uniformed Services Health Education Consortium, San Antonio, USA

**Keywords:** guillain-barré syndreome, demyelinating, polyneuropathy

## Abstract

Acute inflammatory demyelinating polyneuropathy (AIDP), characterized by the autoimmune destruction of Schwann cells with resultant myelin degradation, is the most common subtype of Guillain-Barré Syndrome (GBS). GBS encompasses a myriad of autoimmune polyradiculoneuropathies, typically following an antecedent infectious process. Symptom onset is typically 1-3 weeks following an upper respiratory or gastrointestinal illness and consists of rapidly progressive ascending areflexic motor paralysis. Lower cranial nerves are often involved, leading to bulbar weakness and respiratory compromise. Autonomic dysregulation is common and must be managed carefully to avoid potentially fatal autonomic dysregulation. Contrary to the potential severity of the condition, 66% of GBS cases present with the initial complaint of lower back pain. Intravenous Immunoglobulin (IVIg) and/or plasmapheresis coupled with supportive management is the mainstay of GBS treatment. The majority of patients make a full recovery in up to one year. The rapid and serious nature of the disease coupled with the often benign presentation can make the diagnosis a difficult but vital challenge.

## Introduction

Guillain-Barre Syndrome (GBS) is an immune-mediated polyradiculoneuropathy that is most commonly preceded by a gastrointestinal or upper respiratory illness. GBS can be classified into at least four subtypes. Acute inflammatory demyelinating polyneuropathy (AIDP) is the most common subtype, with autoimmune destruction of Schwann cells leading to myelin degradation of both motor and sensory peripheral nerves. AIDP is the most common subtype seen in North America and Europe. Acute motor axonal neuropathy (AMAN) and acute motor and sensory axonal neuropathy (AMSAN) are more frequently seen in Asia. Miller-Fisher syndrome (MFS) is characterized by the triad of ataxia, ophthalmoplegia and areflexia, and has been known to have clinical overlap with GBS when concomitant limb weakness is observed [1,2]. Symptom onset in AIDP is typically 2-4 weeks following the antecedent illness and consists of acute to subacute progressive ascending areflexic motor paralysis. If lower cranial nerves are involved, bulbar weakness can occur leading to airway compromise and thus require mechanical ventilation. Pain is an often reported associated symptom but is rarely the presenting symptom. More specifically, back pain has a wide range of incidences, with ranges of 13%-62% depending on the study [3]. This has the possibility to delay treatment if overt neurologic symptoms have yet to manifest. We describe a case of GBS-MFS overlap that initially presented as back pain without overt neurologic signs.

## Case presentation

A 57-year-old man with no significant past medical history presented to the emergency department with one week of sharp lower back pain and symmetric lower extremity radiculopathy. He endorsed fatigue, a 10-pound weight loss, as well as fingertip and lip paresthesia’s over the preceding week. Further history elucidated a brief upper respiratory infection several weeks prior. He denied saddle anesthesia, change in bladder or bowel function, fever, night sweats, rash, or recent insect bites. He had visited local urgent care centers twice in the preceding week for his pain and received analgesics and muscle relaxants for symptom relief. Physical exam on admission was notable for symmetrically decreased strength with preserved sensation and reflexes in the lower extremities. Orthostatic hypotension was noted on admission and was responsive to a fluid bolus. Complete blood count (CBC), complete metabolic profile (CMP), creatine kinase (CK), thyroid-stimulating hormone (TSH), urinalysis, and lumbar spine computed tomography were unremarkable. He was admitted for further evaluation and pain control.

His back pain persisted despite analgesia, and he was noted to have progressive lower extremity weakness. On hospital day 3, he developed a left-sided facial nerve deficit consistent with Bell’s palsy and new-onset dysphagia. Magnetic resonance imaging (MRI) of the brain and spine were performed and unremarkable. Lumbar puncture was performed following the MRI and demonstrated albuminocyologic dissociation as evidenced by a cerebral spinal fluid (CSF) sample with 23 nucleated cells and a protein level of 188 mg/dL. A diagnosis of GBS was made, and he was transferred to the intensive care unit for close monitoring of his respiratory status.

A four-day course of intravenous immunoglobulin (IVIg) was initiated at 2 mg/kg, with a total dose of 142 g. Electrodiagnostic studies demonstrated a predominantly demyelinating polyneuropathy affecting both upper and lower extremities. Conduction velocities were slowed and a prolonged F-wave response was noted, findings that were consistent with a diagnosis of AIDP. No denervation changes were noted in his lower extremity musculature, further supporting an acute process of most likely less than two weeks. His motor function improved following the four-day course of IVIg, and he was downgraded when it was deemed he would not require mechanical ventilator support. His back pain gradually improved prior to discharge, and he was ultimately discharged to a rehabilitation facility. Following a short stay at an inpatient rehabilitation facility he ultimately returned home with continued improvement of both strength and pain over the following six months.

## Discussion

GBS is characterized by acute or subacute ascending paralysis and areflexia. The incidence of GBS is rare with a median of 1.11 cases per 100,000 persons per year [4]. Two-thirds of GBS presentations are preceded by an upper respiratory or gastrointestinal illness. The most common infectious etiology is Campylobacter jejuni, constituting approximately 30% of cases [5]. Other common infectious etiologies include Epstein-Barr virus, Varicella-Zoster virus, and Mycoplasma pneumonia [6,7]. If not acutely infected at the time of presentation, a thorough history often reveals an infection within the preceding six weeks. Other preceding triggers include trauma, vaccinations, immunosuppression, pregnancy, and surgery [8]. 

Typical clinical features of GBS include progressive bilateral weakness of the lower and/or upper extremities without overt central nervous system involvement. Reflexes are often diminished and progress until the peak of disease. If a presentation is acute, symptoms progress to a peak in 24 hours. If the presentation is subacute, peak symptoms usually manifest by week 4 of illness. Autonomic dysregulation is common and includes variability in blood pressure, heart rate, temperature, and pupillary dysfunction [8]. Atypical presentations can include asymmetric or predominantly proximal and/or distal neurologic symptoms. Severe pain is also an atypical presentation, with reports of back pain and radiculopathy [2,8]. A multitude of variant presentations are well documented, the most commonly recognized being MFS. Variant presentations rarely present in isolation and are typically seen in overlap with a classic syndrome [2,8].

Diagnosis is based primarily on patient history and physical exam findings, with supporting evidence from CSF and electrodiagnostic studies. The two most common diagnostic criteria are the National Institute of Neurological Disorders and Stroke (NINDS) and Brighton Collaboration guidelines. Each respective criteria was initially devised to study the link between GBS and vaccine administration but has since extrapolated to the diagnosis of GBS from all causes. The criteria aid providers in assessing both typical and atypical presentations, in conjunction with laboratory and electrical studies. Progressive muscle weakness with relative symmetry on both sides of the body along with areflexia or hyporeflexia is a clinical feature requisite for diagnosis. Laboratory studies to evaluate renal, hepatic, hematologic, and endocrine systems are useful to rule out alternative causes of paralysis. CSF studies classically demonstrate elevated protein with a normal cell count, commonly referred to as albuminocytologic dissociation. A common pitfall in the management of GBS is a delay in diagnosis when CSF studies are normal, as CSF protein levels may be normal in up to 50% of patients when assessed in the first week of symptoms. It is therefore recommended that CSF studies be repeated at week 2 when the percentage of patients with normal CSF protein decreases to 10%-30%. Anti-GQ1b antibodies have been reported in up to 90% of MFS, but no other such antibodies are reliably associated with typical or atypical GBS. Electrodiagnostic studies can also aid in the diagnosis, especially when the presentation is atypical. Electrodiagnostics typically show decreased conduction velocity, evoked amplitudes, abnormal temporal dispersion, and/or partial conduction blocks, all of which are evidence of sensorimotor polyradiculoneuropathy. Classic findings include a normal sural sensory nerve action potential with an abnormal or absent medial and ulnar sensory nerve action potential, commonly referred to as a sural sparing pattern [1,8,9].

Several studies have demonstrated that MRI with gadolinium will show enhancement of the cauda equina or nerve roots in patients with acute GBS [10-13]. It has been postulated that this finding can correlate with pain intensity and disease duration. To date, these findings have proven to be sensitive but not specific for acute GBS [10]. As such, the role of imaging in the diagnosis of GBS is primarily relegated to the exclusion of alternative anatomic, infectious, neoplastic, or ischemic etiologies.

The primary treatment of GBS includes supportive care plus IVIg or plasmapheresis. While trials have shown equivalent efficacy between IVIg and plasmapheresis, IVIg is considered first-line therapy due to its ease of administration, widespread availability, and decreased equipment burden [14]. Additionally, trials of combination therapy with plasmapheresis followed by IVIg demonstrated combination therapy is not superior to monotherapy [15]. Due to the potential for respiratory compromise and autonomic instability it is recommended that patients be monitored in a critical care setting. Respiratory status is monitored serially with either negative inspiratory force, maximum inspiratory pressure, or maximum expiratory pressure.

Lower back pain is one of the most common ailments that prompt patients to seek medical attention. The vast majority of cases are benign musculoskeletal pain that resolves with conservative management. A great deal has been made regarding the conservative use of imaging and exhaustive workup as a means to cost-saving and avoiding unnecessary workup [16,17]. Clinicians are well trained to remain vigilant for concerning “red flag” signs that offer clues as to more concerning anatomic, metabolic, or infectious etiologies that may be precipitating back pain. While pain in GBS is well documented, it is often noted to occur as the illness progresses with persistence into recovery. It is atypical and uncommon for back pain to be the heralding symptom of GBS, but has been documented in case series [3]. Our case demonstrates the difficulty of diagnosing GBS when the presentation is early. Additionally, it was only after the variant features consistent with MFS manifested that the diagnosis was made. While back pain is exceedingly common, it is important that providers keep GBS on the differential diagnosis when symptoms persist despite conservative management.

## Conclusions

This case highlights the challenges with diagnosing GBS when the presentation is atypical. GBS is associated with a variety of reported painful symptoms, and can easily be overlooked when the initial workup is unremarkable. Additionally, this case highlights the variant presentations, specifically MFS, which can occur concomitantly with GBS. While anti-GQ1b antibodies were unfortunately not tested in this case, antibodies are often helpful in the diagnosis of MFS when the presentation is atypical.
